# The Role of Social Support in Buffering the Financial Toxicity of Breast Cancer: A Qualitative Study of Patient Experiences

**DOI:** 10.3390/cancers17101712

**Published:** 2025-05-20

**Authors:** Ramona G. Olvera, Sara P. Myers, Alice A. Gaughan, Willi L. Tarver, Sandy Lee, Karen Shiu, Laura J. Rush, Tessa Blevins, Samilia Obeng-Gyasi, Ann Scheck McAlearney

**Affiliations:** 1 CATALYST, Center for the Advancement of Team Science, Analytics, and Systems Thinking in Health Services and Implementation Science Research, College of Medicine, Wexner Medical Center, The Ohio State University, Columbus, OH 43202, USA; rgolvera@gmail.com (R.G.O.); alice.gaughan@osumc.edu (A.A.G.); willi.tarver@osumc.edu (W.L.T.); laura.rush@osumc.edu (L.J.R.); 2 Division of Surgical Oncology, Department of Surgery, College of Medicine, Wexner Medical Center, The Ohio State University, Columbus, OH 43210, USA; sara.myers@osumc.edu (S.P.M.); samilia.obeng-gyasi@osumc.edu (S.O.-G.); 3 Division of Cancer Prevention and Control, Department of Medicine, College of Medicine, Wexner Medical Center, The Ohio State University, Columbus, OH 43210, USA; 4 College of Medicine, University of Cincinnati, Cincinnati, OH 43210, USA; lee6sd@mail.uc.edu; 5 Department of Psychology, College of Arts and Sciences, The Ohio State University, Columbus, OH 43210, USA; tessa.blevins@osumc.edu; 6 Department of Family and Community Medicine, College of Medicine, Wexner Medical Center, The Ohio State University, Columbus, OH 43210, USA

**Keywords:** financial toxicity, breast cancer, qualitative methods

## Abstract

**Background/Objectives**: Financial distress from the direct and indirect costs of cancer treatment is a critical issue for many patients with breast cancer, particularly those from underserved populations who may be more vulnerable to financial hardship and its negative impacts on quality of life and clinical outcomes (i.e., financial toxicity). Few investigations, however, focus on protective factors that safeguard against financial toxicity. This study explores how social support might reduce financial toxicity among patients with breast cancer who are at high risk for financial hardship. **Methods**: We analyzed interviews with 41 adult women treated for stage I-IV breast cancer that had been conducted between December 2021 and March 2022. Our study specifically sampled women considered to be at elevated risk for financial toxicity: young adults aged 18–40 years old, Black women, women with lower incomes, and those residing in rural communities. We used deductive and inductive coding to identify themes related to social support. **Results**: Interviewees reported receiving support from family, friends, and their communities during their treatments. They noted how this social support helped with direct and indirect costs, encouraged emotional wellbeing, and safeguarded against economizing behaviors that offset spending (e.g., financial tradeoffs that jeopardize their treatment plan). **Conclusions**: Patients with breast cancer from groups vulnerable to financial toxicity often rely on the support of family, friends, and their communities to help buffer financial distress from the costs of treatment. These data highlight social support as an area for future studies exploring strategies to mitigate financial toxicity.

## Simple Summary

Financial toxicity is a critical issue for many patients with breast cancer. This qualitative study explores perspectives about how social support influences financial distress among women with stage I-IV breast cancer at risk for experiencing financial toxicity who were treated at a single National Cancer Institute-designated comprehensive cancer center. Participant narratives indicated that social support played a key role in mitigating direct and indirect costs, encouraging emotional wellbeing, and safeguarding against economizing behaviors to offset spending. These data highlight social support as an area for future studies exploring strategies to reduce financial toxicity.

## 1. Introduction

The economic burden of cancer care is a major issue for patients with and survivors of breast cancer [1]. Medical debt incurred during and after treatment can result in psychosocial distress and behaviors that contribute to inferior quality of life and clinical outcomes [2,3,4,5,6,7,8,9,10]. This phenomenon, described as financial toxicity, may be particularly prevalent among underserved demographics such as adolescents and young adults aged 15–39 years at the time of their breast cancer diagnosis, minoritized racial or ethnic groups, those residing in rural communities with limited access to medical care, and individuals with incomes at or near the federal poverty level [11,12,13,14,15,16].

Recognition that these demographics may be especially susceptible to experiencing financial hardship has informed our understanding of financial toxicity. Existing frameworks for financial toxicity synthesize risk factors for experiencing hardship by characterizing the complex interplay between costs of care and patients’ psychological and behavioral responses to the financial burdens of treatment [9,10,17]. Notably, this interplay emphasizes that financial toxicity may occur even among patients with household incomes above the US median or who are well insured [18]. As conceptual models have evolved, increasing emphasis has been placed on the strategies of support-seeking and economizing behaviors to offset treatment expenses [19]. In fact, studies have consistently demonstrated that a lack of social support may worsen patient outcomes. Social isolation, in particular, may affect mental health [20] and treatment nonadherence [21], as well as potentially increase symptom severity [22,23] and mortality [24].

Despite awareness that a lack of social support contributes to financial toxicity, few studies have characterized how the presence of social support can reduce financial distress. As a first step toward understanding how institutional infrastructure can encourage individual support systems as a means of mitigating financial toxicity, we analyzed interviews focused on understanding perspectives about financial hardship among patients with breast cancer who were at high risk for financial toxicity.

## 2. Materials and Methods

### 2.1. Study Population and Design

The study team recruited adult women treated for stage I-IV breast cancer between January 2015 and December 2019 at our National Cancer Institute (NCI)-designated comprehensive cancer center for an interview-based study designed to improve our understanding of the impact of treatment costs on financial toxicity. As existing data indicate that people in certain sociodemographic groups are particularly vulnerable to the economic impacts of cancer care [25], this study employed purposive sampling to select participants from high-risk groups. Specifically, we were interested in the narratives of young adults (YA) (≤40 years old at diagnosis per our institutional definition); Black women; patients residing in rural areas; and women with lower incomes. Black race was classified based on self report. Rural residence was defined based on whether the participant resided in a non-metro county (2013 USDA Rural–Urban Continuum Codes [RUCC]) [26] at the time of diagnosis. Lower income was categorized as having Medicaid coverage for at least one billing encounter. Individuals under 18 years old, those diagnosed with ductal carcinoma in situ (stage 0 disease), patients who were non-English speaking, and those who received consultation without at least 1 modality of treatment at our cancer center were excluded. This study received institutional review board approval (Study# 2020C0174), and the COREQ (Consolidated Criteria for Reporting Qualitative Research) principles informed data collection and analysis [27].

### 2.2. Data Collection and Analysis

After receiving participants’ verbal informed consent, two trained interviewers (LJR and KS) conducted semi-structured interviews by telephone between December 2021 and March 2022. The open-ended questions guided participants to share treatment experiences and discuss direct and indirect costs, resources, barriers, and support (Document S1 in Supplementary Materials). Two researchers (KS and SL) reviewed the verbatim transcripts of the audio-recorded interviews and redacted any inadvertently disclosed identifying information. Three members of the research team (SL, KS, and TB) initially coded interview transcripts using deductive dominant content analysis [28,29]. Two team members (RGO and AAG) inductively analyzed codes related to descriptions of social support [30]. During analysis, we refined the definition of social support to include any material or emotional support [31] provided by family, friends, or community members or institutions (e.g., cancer centers or community-based non-profit organizations) that targeted the costs of care. The research team classified themes and subthemes based on participants’ narratives, informed by existing frameworks for financial toxicity [9,10,17,19]. An iterative coding process, supported by the use of the NVivo (version 12) software (Lumivero, Denver, CO, USA), facilitated discussions among the research team until we achieved consensus about codes and themes.

## 3. Results

**Figure 1:**
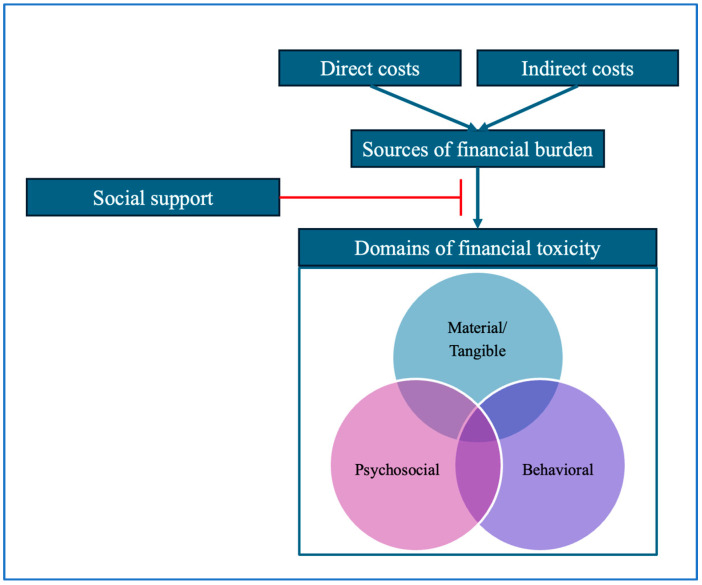
Proposed framework presenting modifiable factors for financial toxicity. While direct and indirect costs serve as sources of financial burden and perpetuate financial toxicity, social support may have a protective effect (denoted as a red T-shaped line). Financial toxicity is represented as a multidimensional domain and adapted from the Financial Hardship Typology created by Altice et al., 2017 [10].

Of the 41 participants, 39% were YAs, 34% self-identified as Black, 34% were rural-residing, and 32% met the study criteria for low income. The average age of participants at diagnosis was 45 years old (SD 10; range, 26–73). Participant narratives revealed that themes of support closely adhered to the material/tangible, psychosocial, and behavioral domains of financial toxicity typified in existing frameworks, leading us to propose the integrated framework we present as Figure 1. Next, we describe the social support participants received from family, friends, and the community by the three domains of financial toxicity. We provide tables with additional representative quotes in support of these characterizations. 

### 3.1. Support in the Material/Tangible Domain

Participants described material and tangible forms of support that they reported offset direct and indirect costs of care (Table 1). These included monetary and non-monetary support to assist with copays related to treatment, transportation to and from medical appointments, childcare, and assistance with paying household bills or accomplishing household tasks. Participants reported that financial assistance from friends was helpful in bridging interruptions in insurance coverage. One YA patient highlighted how her friends leveraged social media to cover costs that resulted directly from medical expenses and indirectly from lost income related to absence from work.

*[Friends] set up a GoFundMe because I had an issue with my insurance and they… did a GoFundMe to like help cover any time off work and any procedures I needed and stuff like that.*

Covering the cost of gas or having a supportive individual drive them to their appointment(s) was also reported as beneficial. A participant living in a rural area stated the following:

*I was able to get [to appointments] in a decent amount of time, but it did take a toll with gas. However, I had some really good people, some really supportive people in my life and my staff … took up a collection and gave me a huge gift card. That was just unbelievable. And I was able to use that for a lot of my gas expense.*

Although many recounted the generosity of family, friends, or community acquaintances, some participants commended services provided by community-based non-profit organizations. Multiple women described receiving services from the Pink Ribbon Girls (currently known as the Pink Ribbon Good), similar to this participant:

*The Pink Ribbon Girls, they really stepped up and they took me to most all of my appointments. They even took me to one of my surgeries, you know, at like five something in the morning.*

**Table 1:** Buffering material/tangible responses to financial burden through social support.

Support System	Participant Quotes
Family	“I never worried … because my dad’s helping me out. But I knew my bills were going to be paid and my dad, he filled in those gaps between the disability and my job. And then if I needed something in between. And I will say my son’s father did step up to the plate, too.”
	“My other sister, she would come and get me and take me to the pharmacy to pick up my prescriptions and stuff like that.”
Friends	“They [friends] collected money for me, they collected over $670. That paid my rent, my internet bill, put food in my house, and left me with money.”
	“Like, one of my friends paid my cell phone bill for a couple months, which was amazing. And like I said, without their help, it would have been a lot harder. And I felt really bad that they did that because I’m pretty proud, you know. But it was amazing and let me know how much they care that they did do it.”
Community	“I do recall maybe last year or the year before, they did a program at [the cancer center] and they did assist me with my medicine. … They helped me out paying for my medicine. So that helped me out so much.”
	“I was fortunate in that [I am] someone who knows a lot of people and who’s known a lot of people in the community. I mean, I had GoFundMes and stuff like that. I would have lost my house, would have been homeless, without a doubt, without community support.”

### 3.2. Social Support in the Psychosocial Domain

Alongside their discussions of material support, participants described how these acts of support impacted their emotional responses and distress (Table 2). The interviewees noted the positive effects of sentimental displays (e.g., cards). As one woman noted, *“when you’re having like a sad day, it is nice to reflect on that, and the amount of people that just reached out and sent me cards and things…”*

Participant narratives showed how acts of kindness could lessen the financial and emotional burdens of cancer treatments, with one woman stating the following:

*…being able to have those meals available or gift cards to help out. I mean, just those little day-to-days that we take for granted, they become a bigger deal when you’re struggling with that diagnosis and trying to get through surgeries.*

Another suggested that supportive interactions affirmed her religious faith:

*I was living off the grace of God, by God sending me people in my life, just different strangers. They helped me get by. And then my job started to, my co-workers were like doing a fundraiser for me, so they would go around and do fundraisers. Like, hey, we try to raise money for [participant’s name]…*

These types of support allowed patients to feel a sense of normalcy during tumultuous periods navigating their cancer diagnosis and treatment. For example, a participant described the opportunity to “go have fun” because of the unexpected support from others.

*It was surprising how I would get just cards in the mail occasionally, just out of the blue, and that was something that was just so sweet. Sometimes, you know, we get like a gift card for gas or, you know, a restaurant. One time we got one for, you know, Dairy Queen. Just, you know, it just said, go get some ice cream and have fun … That and often it was from people… I never would have thought would send something to us.*

**Table 2:** Buffering psychosocial responses to financial burden through social support.

Support System	Participant Quotes
Family	“She’d [mother] come over and make sure that I had something to eat. And you know, just basically checking in on me, and just making sure that I’m okay.”
Friends	“[Friends] took care of [my son] for me and they wouldn’t let me pay them either. I had a great support system, I don’t know what I would have done without them though, I don’t know how I would have made it through.”
Community	“We live in a pretty amazing community where I had a lot of food and a lot of gift cards given to us. So, that burden was lifted for a period of time and in a really, really beautiful way.”

### 3.3. Effects of Social Support on the Behavioral Domain

Participants identified how financial assistance allowed them to avoid economizing behaviors to offset costs (Table 3). One participant indicated that transportation barriers contributed to nonadherence to her oncology appointments; family support averted this challenge.

*His [husband’s] sister was a big, big help, like with the kids. And she loaned us $10 or $20 here and there, just to get us through. My mom and dad would loan us $20, $30 to get us through. We had a little bit of support here and there. But there was times where I just couldn’t make my appointment because I didn’t have the gas to go; I’d have to change my appointment day.*

Another described family support that allowed her to avoid lost income from time off from work and from non-medical costs related to travel and lodging.

*My son lives [near the cancer center]…, and I’m able to work remote for my job. So, I had a lot of luxuries that I know a lot of people don’t have—where I stayed with him the whole week, and I worked from his place, and I went and had my treatments and went back to his place. So, I was, I was pretty fortunate where I didn’t have to worry about paying for room and board, and gas, and all that. Probably not everyone has that, unfortunately.*

**Table 3:** Buffering behavioral responses to financial burden through social support.

Support System	Participant Quotes
Family	“I planned my chemo days around [my husband] because he works, he gets two days off…. He is set in stone like he knows exactly where he’s going every day. He goes to the same stops at the same time every day, like he has his routine. And I didn’t want him to up end any of that, you know, because I was thinking of his needs. He was like, ‘I’m thinking of your needs.’ I’m thinking of his needs…I went to the [the cancer center’s] schedule arm. Like, ‘I don’t care what time, what day as long as it’s on a Thursday. Can we make all of my chemo on a Thursday?’ And the scheduler said, ‘Yeah, I think we can do that.’”
Friends	“Just friends who came to visit, wrote cards, sent care packages, that kind of thing… My friend from Germany came and my friend from Korea and I don’t think that they could pick Ohio on a map. And so just like having people care enough to be there with you and be in the thick of it was just so comforting. And it just helped me get kind of through everything, because looking uphill at … 16 chemo treatments, just everything. You kind of are, like, am I ever gonna get through that?”
Community	“And there was an [academic medical center] thing for Christmas gifts for my daughter, I forget what it’s called. I think it’s through this, [the cancer center] is how I found out about it. Like, they were like, oh, you should apply for this and while you’re going through like active surgery or treatment, they provide like Christmas gifts for your family…That was a really great program.”

## 4. Discussion

In this analysis of interviews with lower-income, YA, Black, and/or rural-residing women with breast cancer, participants shared how material and tangible support from family, friends, community members, and non-profit groups was instrumental in alleviating financial distress. Participants described social support that lessened the impact of both direct and indirect costs of care, may have provided psychological benefits, and reduced their economizing behaviors. The overlapping themes identified in this study suggest that robust social support may mitigate factors in the domains contributing to financial toxicity (Figure 1) [10].

Participants discussed how support helped reduce direct and indirect costs contributing to financial distress. Direct costs included expenses related to treatments and medications but also non-medical costs associated with being able to attend appointments, such as the costs of transportation to and from a treatment facility and childcare. Indirect costs included lost income because of reduced work hours, which translated into concerns over being able to pay for essentials such as utilities, housing, and food. Existing interventions to reduce the impact of direct and indirect costs on financial toxicity include financial navigation, a structured approach to identifying sources of financial hardship, assisting with applications for financial assistance, and tracking patient-reported outcomes over time [32].

Unconditional cash transfers and grants from non-profit organizations have also shown some benefits [33]. Eligibility for these opportunities and access to resources, however, vary and may exacerbate disparities in care among those with increased vulnerability. For example, although most NCI-designated cancer centers report offering financial services, institutional resource barriers may limit the availability of services and the capacity to deliver assistance to those who need it [34]. Patients living in rural settings who must travel prohibitively long distances to be treated at NCI-designated centers [35] and those not captured by targeted financial screening (e.g., YAs) [36] may have few prospects for aid. Our study highlights how individuals in these circumstances may instead turn to crowdfunding sources such as GoFundMe to reduce financial hardship. These data corroborate recent investigations demonstrating the efficacy of crowdfunding platforms in reducing financial toxicity and logistical burdens among those with pediatric and gynecologic cancers [37,38]. Using interventions that leverage social media and personal relationships as ways of expanding options for assistance may be a supportive strategy to reduce long-term cancer-related financial hardship, but further investigations are needed to understand the variable success of crowdsourcing campaigns among certain populations [39]. For example, studies have reported that although social support for Black women during breast cancer treatment is high [40], GoFundMe campaigns may be less fruitful for Black fundraisers [41]. Exploring the potential of these interventions, as well as targeted social assistance and community support, in mitigating financial toxicity will be an important direction for future work.

Although psychological distress resulting from medical debt is a well-recognized component of financial toxicity [42], few studies have shown how psychological support linked to material/tangible assistance impacts patients. Participants in our study endorsed feelings of gratitude for the compassion and thoughtfulness conveyed through cards, notes, and visits from members of their support networks. Psychosocial support plays an integral role in reducing distress, financial or otherwise, among patients with cancer, and has been associated with improved quality of life and survival outcomes [43]. It has also been shown to improve coping among patients with cancer [44,45]. Thus, we postulate that psychosocial support may reduce financial hardship via multiple mechanisms.

Reducing spending on essentials like medical care, food, and clothing is another well-documented coping behavior exhibited by patients with cancer to offset costs [46,47]. In our study, monetary and non-monetary social support provided some women with additional coping mechanisms, reducing their need for further behavioral changes. These findings are similar to a study that reported that younger and lower-income patients with cancer were more likely to alter treatment plans and lifestyles to cope with financial distress [46].

Our study has several limitations that should be noted. The sample was drawn from one academic cancer center in a Midwestern city in the United States, which may not capture the experiences of patients with breast cancer from other regions or those receiving different financial support services at other types of institutions. This study did not assess objective metrics of material strain or financial hardship, such as precise costs of care, household income, or competing sources of medical debt that may have preceded and exacerbated costs of cancer treatment. Additionally, we completed our interviews with survivors of breast cancer who spoke English, thereby excluding the experiences of non-English speakers who may receive different types of support. Due to the timing of the interviews, some of the participants may have experienced social isolation during their cancer treatment due to the COVID-19 pandemic, thus limiting their access to social support. These data may be further challenged by participants’ difficulties recalling their experiences if their treatments were remote, as well as due to changes in healthcare reform, insurance reimbursements, etc., that may have occurred since the data were collected. This study captures perspectives at a single point in time and does not address how financial toxicity might evolve during and after treatment. Future research should target other populations to expand on our study, including individuals with different types of cancer and from different regions. It is important to acknowledge that not all our participants mentioned receiving social support that alleviated financial distress, and the amounts of financial and non-monetary social support participants reported receiving varied. Of note, relying on social networks to help buffer financial toxicity may exacerbate differences among groups that already experience health disparities.

## 5. Conclusions

Family, friends, and community partners provide financial and social support to patients with cancer. Participants in our study reflected on the assistance they received for direct and indirect cancer-related costs, indicating that such support may be crucial to treatment adherence, coping, and financial wellbeing. Future investigations are needed to understand how to best leverage these sources of support and evaluate the utility of programming to increase social support as a strategy to reduce financial toxicity.

## Data Availability

The data are unavailable due to the privacy of the participants.

## Supplementary Materials

The following supporting information can be downloaded at https://www.mdpi.com/article/10.3390/cancers17101712/s1, Document S1: Interview Guide.

## Abbreviations

COREQ	Consolidated Criteria for Reporting Qualitative Research
NCI	National Cancer Institute
RUCC	Rural–Urban Continuum Codes
YA	Young adults
