# Supplementary material for: The Role of Social Support in Buffering the Financial Toxicity of Breast Cancer: A Qualitative Study of Patient Experiences
Source: Cancers (Basel). 2025 May 20;17(10):1712. doi: 10.3390/cancers17101712 (PMC12110526; doi:10.3390/cancers17101712)
Supplement: Supplementary file 1 [file cancers-17-01712-s001.zip › cancers-3616709-supplementary.pdf]

Financial Toxicity and Vulnerable Breast Cancer Populations

Patient Interview Guide - Combined

Introduction and Background

First, let me THANK YOU for agreeing to participate in this research project. I am (select as necessary)

- [**INSERT NAME OF RESEARCHER**] and I am a researcher with the Ohio State University in Columbus, Ohio.
- [**INSERT NAME OF RESEARCHER**], a surgical oncologist in the Division of Surgical Oncology at the Ohio State University in Columbus, Ohio.
- [**INSERT NAME OF RESEARCHER**] from the Recruitment, Intervention and Survey Shared Resource Center at the Ohio State University in Columbus, Ohio.

I/We are here to learn from you about the experiences you have had with breast cancer treatments and the costs of those treatments.

You have been identified because you have been diagnosed with and treated for breast cancer and we want to learn from you about how receiving breast cancer treatment has affected your personal and family finances. We have scheduled the next hour to ask you questions about this subject and related issues. Please note that we recognize that some of these questions may be sensitive, for example, how the costs of cancer treatment have affected your family, and you are welcome to take a break from the interview whenever you want.

Interview Overview

As an overview, I/we have several general topics we will be asking about:

1. Background: Information About Treatment
2. Costs of Cancer Treatment
3. Financial Barriers to Treatment and Barriers to Accessing Financial Resources
4. Attitudes Towards Accessing Financial Support for Treatment Costs

We have scheduled the next 60 minutes to discuss these topics with you.

Informed Consent

Before we begin the interview, we need to review our informed consent process. Prior to today, you were sent the informed consent document explaining our study, and the benefits and risks to you of participating. Before we begin this interview, let me make sure that you understand that:

a. Your participation is completely voluntary. If you do choose to participate, you may end the interview at any time.
b. We consider this discussion to be confidential. Your participation is confidential in the sense that your name will not be used in any reports or articles.
c. We would also like to record the interview for the purposes of data collection for our research. The recording will not be used to identify you in any way.

Do you have any questions about our study or this interview process?

Interview Questions

Domain 1: Background: Information About Treatment

- To get started, could you provide an overview of your experience with your breast cancer diagnosis, treatments, and follow-up?
- What types of health care providers (e.g., clinical oncologists, surgical oncologists, radiation oncologists) were involved in helping you decide what type of cancer treatment to get?
- What questions did you ask your health care provider regarding cancer treatment costs? [**Probe**: Were your questions answered to your satisfaction?]
- Did any of your health care providers talk about your financial situation before helping you to decide about a treatment plan?
- What types of treatment (e.g., chemotherapy, surgery, radiation therapy) did you receive at the Spielman Center?
- How long have you been coming to the Spielman Center? [**Probe**: Was your first visit before or after you were diagnosed with breast cancer?]
- How did you get to the Spielman Center when you went for treatment? Did anyone else go with you? Who were they?
- How difficult is it to get to the Spielman Center for any treatment or follow-up care? [**Probe**: What makes it challenging?]
- How has the COVID-19 pandemic changed how you get your cancer treatments and/or follow-up care? Please describe.
- Do you have any concerns about the long-term impact of the COVID-19 pandemic on your cancer treatments and/or follow-up care?

Domain 2: Costs of Cancer Treatment

- How much was cost a factor in deciding which treatment to get (e.g., radiation, surgery, and/or chemotherapy)?
    - Were you given any information about the costs of cancer treatment options before you made your decision?

- What type of health insurance have you had throughout your treatment, if any?
    - Has your insurance coverage changed at any point during your treatment? [**Probe**: If so, can you please explain why?]

- If the person HAS INSURANCE: Were there any aspects of your cancer treatment that were not covered financially by your insurance? Do you know why they were not covered by your insurance?
    - Were you surprised by any of the co-pays, deductibles, or premiums you had to pay during your cancer treatment or as part of follow-up care?

- If the person DID NOT HAVE INSURANCE AT ANY POINT: What aspects of your cancer treatment do you wish you had help paying for? [**Probe**: Were there others that you would have liked help paying for as well that weren't direct costs (e.g., transportation, childcare)?]

- What types of things did you have to pay for out-of-pocket during your treatment that was not related specifically to your medical care (e.g., co-pays, co-insurance, etc.) but were still due to your need to get care (e.g., transportation, lodging, childcare, additional home services)?

- At any point during your treatment(s) did you think that money was getting tight? [**Probe**: Do you have any stories about this you could share?]

- Have you ever had to skip treatments because they were too expensive? [**Probe**: Do you have any stories about this you could share?]

- Did your diagnosis or the treatment process affect your ability to work or continue working? [**Probe**: If yes, could you please describe?]

- How did your diagnosis and/or treatments affect your personal and/or family finances?
    - What adjustments, if any, have you had to make based on the cost of your cancer care (e.g., moved to a new home, not participating in activities, etc.)?

Domain 3: Financial Barriers to Treatment and Barriers to Accessing Financial Resources

- Knowing what you know now about the costs of your cancer treatment, would you change any of the decisions you made about your treatment or the care you received?

- Is there anything you are still paying for out-of-pocket that you didn't expect to still be paying?

- Do you have medical debt due to your cancer treatment?
    - Does that debt affect whether or not you get your treatment? [**Probe**: If yes, could you please describe how?]
    - What types of bills are you still paying off?

- Can you talk about what challenges you faced trying to find financial support during your cancer treatment?

- If there was a time during your treatment when money was tight, who did you turn to for help with costs such as paying copays, coinsurance, for other treatment costs? [**Probe**: why did you turn there?]

- <u>If you had trouble getting help covering the costs</u> of your cancer treatment, what were the biggest challenges? Can you please provide an example?

- <u>If you did not have trouble getting help covering the costs</u> of your cancer treatment, what things made it easy for you?

- Are there any friends or family members who helped you financially during your cancer treatment? In what ways did they support you?

Domain 4: Attitudes Towards Accessing Financial Support for Treatment Costs

- Have you been offered financial assistance or payment options by your health care provider or insurance company? [**If yes,** could you please explain?]

- Have you ever applied for any financial assistance to help with your cancer treatment (e.g., hospital, community, state)?
    - If yes, what was that process like?
    - If no, have you ever considered applying for assistance? Why or why not?
    - If there is anything you would change about that process, what would it be?

- Is there any type of information you wish you had received about how to get assistance to pay for breast cancer treatments?

- Do you or have you worked with a patient navigator, care coordinator, social worker or financial counselor during your treatment?
    - What was that experience like?
    - Was it helpful? Can you please explain?

- Looking back, is there other support or assistance that you wish you had that could have helped reduce the financial burden of cancer treatment for you and/or your family?

Domain 5: SUPPLEMENTAL DOMAINS, BY INTERVIEW POPULATION:

5a. African American Patient-Specific Questions ONLY

- Do you think any of your providers treated you differently compared to other patients? If yes, how so?

- Were there times during your treatment where you experienced discrimination? Can you please describe?

5b. Rural Patient-Specific Questions ONLY

- What is the most difficult part about having to travel to Ohio State University for treatment?

- Did you make housing accommodations in order to be closer to Ohio State University to get treatment?
    - If yes, can you please explain how those accommodations were made and what you learned from that experience?
    - If no, why didn't you?

- At any point did you consider getting treatment closer to home? [**Probe**: Why or why not?]

5c. Medicaid/Low-Income Patient-Specific Questions ONLY

- Has the cost of your cancer treatment(s) impacted other expenses for your family?

- Have you ever had to skip treatments because they were too expensive?

- Did you have difficulty paying for transportation, gas, utilities, rent/mortgage or other bills during your treatment?

- Were there any other cost not covered by Medicaid that you would like to discuss?

5d. Under-40 Patient-Specific Questions ONLY

- What financial hardships do you think stand out the most for women who have breast cancer and are under age 40?

- What do you think will be the long-term impact of these financial hardships?

INTERVIEW CLOSURE AND FOLLOW-UP

- If you had a magic wand, what are the main things you wish had been different during your cancer treatment(s)?

- Is there anything else we should consider that you would like to share with us?

THANK YOU!! so much for your time and participation. Your comments were extremely helpful.
